# The importance of methodology to palliative care research: A new article type for Palliative Medicine

**DOI:** 10.1177/02692163211069566

**Published:** 2021-12-29

**Authors:** Jenny T van der Steen, Melissa J Bloomer, Sandra Martins Pereira

**Affiliations:** 1 Department of Public Health and Primary Care, Leiden University Medical Center, Leiden, The Netherlands; 2 Department of Primary and Community Care, Radboud university medical center, Nijmegen, The Netherlands; 3 School of Nursing and Midwifery, Deakin University, Geelong, VIC, Australia; 4 Centre for Quality and Patient Safety Research, Institute for Health Transformation, Deakin University, Geelong, VIC, Australia; 5 CEGE: Research Center in Management and Economics, Universidade Católica Portuguesa, Porto, Portugal; 6 Instituto de Bioética, Universidade Católica Portuguesa, Porto, Portugal

Palliative Medicine now welcomes submissions of ‘Research Methodology and Methods.’ This comes after a recent editorial board meeting where the question was raised, ‘Do we need a palliative-care-research methodology type of article?’ As a research-oriented journal, Palliative Medicine aims to publish about high-quality research characterised by scientific excellence and ethical soundness, with implications for palliative care clinical practice, policy, theory and methodological knowledge. Therefore, we recognise the importance and impact of articles focussed on novel approaches to research design and on improving research methodologies and instruments as essential to Palliative Medicine and its readership.

One might believe methodological papers to have less impact because they are more likely to be used by researchers only, rather than by a readership of researchers, practitioners, educators and policy makers. However, Leahey et al.^1^ found that new results and new theories tend to reinforce prior work (consolidate knowledge flows), whereas it is new methods that tend to detract from the foundational work that gave rise to them (disrupt knowledge flows). Novel methods are impactful also because they are often imported from other fields, and quickly absorbed into non-methodological writings.

The palliative care community has seen major advances in the theory, design, methods, ethics, reporting and dissemination of palliative care research, since Cicely Saunders, in her 2001 article on the evolution of palliative care, remarked that ‘we were too slow in establishing full academic rigour (and we still have some way to go)’.^2^ Today, our mission to achieve rigour and evidence within the field has not ended and we feel that an important way to professionalise and build upon previous work is to publish on methodological advances. In fact, the growth of people living with serious illnesses has not been accompanied by the same advancement in evidence on how to care for them. Evidence growth has been impeded by small, single-site trials, unrepresentative study cohorts and studies lacking sufficient rigour.^3^ By inviting submissions focussed on ‘Research Methodology and Methods,’ we acknowledge its importance to strengthening future research that informs palliative care development, while offering flexibility where it would be challenging to fit the advancement into an existing paper style such as an original article or systematically constructed review.

Palliative care research is complex and diverse. For instance, it includes research focussed on recognising patients’ values, wishes and goals of care, managing biopsychosocial symptoms and spiritual problems, improving quality of life, communication and decision-making and health professional teamwork; all of these within the context of serious, life-threatening or life-limiting illnesses.^4,5^ To perform high-quality ethically-sound palliative care research, a wide range of methodologies and approaches is needed. This requires input from a variety of disciplines such as bioethics and research ethics, biostatistics and epidemiology, humanities, education, economics, e-Health, health services research and implementation science.^4,5^ Such variety risks that we are not benefitting enough from the specialist input needed to optimise design and conduct of palliative care research. Also, we need rigorous methodological research to ensure that palliative care research includes the views and experiences of all involved: patients, their families and loved ones, healthcare professionals and teams, organisations, policymakers, communities and societies.

In this editorial, we address in more detail methods to increase patient and public involvement, and methods of two types of design that are of particular relevance to help increase the evidence-base in the field of palliative care: trials and consensus group methods, which can also be used for methodological issues. Therefore, improving methods and reporting on trials and Delphi-type of studies to address the specific challenges encountered for research in our field will be particularly effective in moving the field forward.

Trials on effects of palliative care have been included in recent systematic reviews such as the Cochrane review on the effects of hospital-based palliative care.^6^ Consistent with previous reviews, the authors conclude that the quality of the evidence is low to very low. The quality of the evidence is often downgraded due to lack of blinding, but a meta-epidemiological study on Cochrane reviews found no different effect by blinding, indicating that blinding is less of a concern than often believed.^7^ A complex intervention is not easily standardised, but the quality of the evidence could be improved if samples were larger, patient populations and models of service delivery were more homogeneous. This calls for better reproducibility of findings, increasing rigour through established, standardised methods and solutions to achieve standardisation.^8^ However, innovation is needed to move beyond the standard trial design, for example, assessing how to optimally design it as either a pragmatic or an explanatory trial.^9^ This requires considering the stadium of the evidence-base. For instance, if developers of an intervention have demonstrated effectiveness, do we, with a more pragmatic approach, expect a diluted effect, or a similar effect when adapted to a different context? Refining theory on how the intervention may or may not work and for whom, is helpful to this end. Sophisticated designs may include pre-planned mediation, predefined subgroup or exploratory tree-based subgroup analyses for which we, however, often need large sample sizes. Mixed-methods designs with increased integration of various quantitative and qualitative methods can also help address questions on mechanisms and understand effectiveness for patients with relevant, particular characteristics. Various types of mixing different methods are often helpful to generate evidence on how interventions were delivered in practice and perceived by patients, families, caregivers and other stakeholders.^10^

Researchers increasingly need to pro-actively consider research design and any innovations to maximise the impact of their research. Even though this is a highly creative process, reporting guidelines, which are a requirement for submission to Palliative Medicine, could also be helpful in preparing a study protocol, and in reporting of ethical approvals, transparency and rigour. Jünger et al.^11^ for example, created a reporting guideline for Delphi studies in palliative care, which combines research evidence with expert consensus to inform service development, clinical practice, policy and further research in palliative care. In this issue of Palliative Medicine, Hussain et al.^12^ used nominal group techniques to achieve consensus on guidelines for management of missing values in datasets.

Reporting guidelines also exist for involving patients and public in research.^13^ Patient and public involvement is another important area in which methodological innovation is particularly welcomed to increase relevance of palliative care research. Globally, the strong emphasis on patient and public involvement in all stages of the research process, including identifying research priorities, contributing to study design, data collection and analysis, dissemination and implementation of research findings,^14^ recognises the potential to improve the relevance, quality and impact of research.^13^ Palliative care research should be no different, but it is essential to acknowledge long-standing assumptions and judge-values that undertaking research with people receiving palliative care or who are dying is improper and unethical. However, patient vulnerability should not be universally assumed and should not prevent from research participation.^5^ Rather, persons who are dying should be afforded an opportunity to participate in research, as they are able and willing, in order to help others and contribute to science that improves care.^15^

In addition to clinician gatekeeping when recruiting for research participation during sensitive consultations, palliative and end-of-life research can encounter multiple ethical challenges in conducting research with vulnerable populations.^5,16–18^ High-quality ethically-sound methodologies in palliative care research are needed to protect individuals but also to ensure that research is conducted in a way that serves interests of individuals, groups, and society at large.^5,19,20^ To increase relevance to groups and society, patient and public can be involved in palliative care research, yet there is limited evidence to describe the best approaches for populations affected by life-limiting illnesses.^14^ There are new challenges involving them in big data research, which has gained significant research momentum.^21^ An assumption that big data research in palliative care is too scientific has presented an additional a barrier.^22^ To address this, starting with humanising the big data, and demonstrating its potential to be meaningful is key to patients, carers and the public getting involved.^14^

The culture surrounding palliative care research is changing, leading to new ways of thinking about and approaching research, such as fostering collaborative, interdisciplinary and intersectoral approaches and diversity among those involved in the research process.^8^ Palliative care research has an essential role in informing and influencing evidence-based clinical practice, service development, education and policy.^23,24^ The soundness of methodological and ethical procedures is therefore paramount. In this editorial, we have highlighted the rationale and key elements for offering the new type of article ‘Research Methodology and Methods.’ Further guidance and author instructions are on our website. We warmly welcome approaches to discuss submissions or seek any further clarifications.
